# Adherence to Daily Food Guides Is Associated with Lower Risk of Metabolic Syndrome: The Nutrition and Health Survey in Taiwan

**DOI:** 10.3390/nu12102955

**Published:** 2020-09-27

**Authors:** Ming-Chieh Li, Hsin-Yu Fang

**Affiliations:** Department of Public Health, China Medical University College of Public Health, Taichung 406040, Taiwan; u106026494@cmu.edu.tw

**Keywords:** metabolic syndrome, NAHSIT, daily food guides, dietary guidelines

## Abstract

Although nutritional health knowledge serves as the basis for the daily food guides, limited epidemiologic studies were conducted to verify whether adherence to the daily food guides reduced the prevalence of diseases. This study aims to examine whether adherence to the daily food guides relates to the lower risk of having metabolic syndrome, as well as to assess the association between levels of adherence to daily food guides and demographic characteristics. A cross-sectional study was conducted using data from the Nutrition and Health Survey in Taiwan (NAHSIT) 2013–2016. Face-to-face dietary assessments were conducted using a validated food-frequency questionnaire. Six food groups were defined according to the daily food guides in Taiwan. We constructed a daily food guide index to measure the levels of adherence to the daily food guides. Logistic regression was performed to assess the association between the levels of adherence to the daily food guides and the risk of having metabolic syndrome. A total of 2534 participants (51% of females) were included in the final analysis. After adjusting for age, sex, body mass index, education level, marital status, and family income, we found a negative correlation between the levels of adherence to daily food guides and the prevalence of metabolic syndrome. The odds ratios (ORs) for the highest versus lowest quartile of the adherence level was 0.65 (95% confidence interval (CI) = 0.48–0.88). In addition, males, younger age, lower education, divorced, separated, and widowed, and lower family income were associated with lower adherence to daily food guides. In conclusion, participants reporting better adherence to the daily food guides during the past month had a lower risk of having metabolic syndrome.

## 1. Introduction

The metabolic syndrome is a major predictor for cardiovascular disease, which consists of risk factors including hypertension, obesity, dyslipidemia, and impaired glucose metabolism [1,2]. Several studies have found that dietary patterns were associated with metabolic syndrome. For example, Williams et al. published the very first study describing the association of dietary patterns with metabolic syndrome [3]. They found that the healthy balanced dietary pattern, defined as frequent intakes of raw and salad vegetables, fruits in both summer and winter, fish, pasta, and rice and low intake of fried foods, sausages, fried fish, and potatoes, showed protective effects on the risk of central obesity, elevated fasting plasma glucose, 120 min non-esterified fatty acid and triacylglycerol. Several studies found that people with certain dietary patterns had greater chances of developing metabolic syndrome. A multicenter population-based prospective cohort study conducted in the US found that high intakes of refined grains, processed meat, fried foods, and red meat were positively associated with the risk of developing metabolic syndrome [4]. The Framingham Study has shown that women consuming a dietary pattern with the highest amounts of “empty calories” had the highest risk of metabolic syndrome [5]. A study in the Japanese population found that the western dietary pattern (high in sweets, butter, soda, mayonnaise, sugar, cookies, the tail of a lamb, hydrogenated fat, and eggs) might be associated with increased triacylglycerol and blood pressure On the contrary, a prudent pattern (high in fish, peas, honey, nuts, juice, dry fruits, vegetable oil, liver and organic meat, and coconuts and low in hydrogenated fat and non-leafy vegetables) was associated with reduced risk of an abnormal fasting blood glucose level [6]. The ATTICA study in Greece found that a dietary pattern that includes cereals, fish, legumes, vegetables, and fruits was independently associated with reduced levels of clinical and biological markers linked to the metabolic syndrome, whereas meat and alcohol intake showed the opposite results [7]. Reduced intake of processed foods high in refined grains and adherence to a neo-traditional eating pattern characterized by plant-based fiber, seafood, and coconut products may help to prevent the increasing prevalence of metabolic syndrome in the Samoan islands [8].

Despite that adapting certain dietary patterns might be good for reducing the risk of having metabolic syndrome, policymakers, including the government in Taiwan, seldom declare the adoption of one or the other. Instead, a universal dietary guideline or daily food guides were more often used when making policy. Although nutritional health knowledge serves as the basis for dietary guidelines and the daily food guides [9], no or limited epidemiologic study was conducted to verify whether adherence to the dietary guidelines will relate to or reduce the prevalence of diseases in Taiwan. Some studies in Taiwan have identified the possible dietary pattern that is related to health outcomes [10,11,12], but it is still not clear how adherence to the dietary guidelines or the daily food guides relates to disease outcomes. It is possible that the recommendations in total may not be beneficial for some diseases because the dietary guidelines were designed for the prevention of an array of chronic diseases [13], resulting in the inclusion of recommendations that may be protective for some diseases but not for others. In fact, some people have argued that dietary guidelines may do more harm than good [14,15].

Therefore, the aim of this study is to examine whether adherence to the daily food guides relates to the risk of metabolic syndrome. In addition, we will also identify the factors that were associated with the levels of adherence to daily food guides.

## 2. Materials and Methods

A retrospective cross-sectional study was conducted using data from the Nutrition and Health Survey in Taiwan (NAHSIT) 2013–2016. NAHSIT is a nationwide representative survey aimed to investigate and monitor the nutritional status of Taiwanese people. The survey method has been described elsewhere [10,11]. Briefly, Three-stage probability sampling was used to select the study samples within each stratum. The first stage was the selection of eight primary sampling units (townships and city districts) using probability proportional to size sampling methods. A total of 160 townships or city districts were chosen. The second stage was to randomly select several starting households to construct sampling clusters within each selected primary sampling units. In the third stage, door-to-door visits were conducted to carry out interviews until the required number of the age and sex groups was reached. To avoid seasonal variations that may affect dietary consumption and nutritional status, the Latin square design was used to allocate data collection times evenly over the four seasons. Study participants of NAHSIT were non-institutionalized Taiwanese nationals. Demographic data including sociodemographic characteristics (age, sex, education, and marital status), lifestyle factors (smoking, alcohol drinking, and regular exercise), and self-reported medical history were obtained through face to face interviews. Participants were invited to attend a physical examination at a temporary health examination station.

The study sample will be restricted to adults aged 19 to 64 according to the definition of adults adapted from the newest version of daily food guides and also to avoid bias due to age factors. This study was approved by the China Medical University & Hospital Research Ethics Center (CRREC-108-136). All participants originally gave informed consent to participate in the Nutrition and Health Survey in Taiwan including consent for their data to be used for future research purposes.

### 2.1. Metabolic Syndrome

Metabolic syndrome is defined by the presence of any three of the following five conditions [16]: (1) waist circumference ≥ 90 cm for men and ≥ 80 cm for women, (2) systolic blood pressure ≥ 130 mmHg and diastolic blood pressure ≥ 85 mmHg, (3) HDL-C < 40 mg/dL for males and 50 mg/dL for females, (4) fasting plasma glucose level ≥ 100 mg/dL or history of antidiabetic treatment, and (5) triglyceride ≥ 150 mg/dL or medication use.

### 2.2. Measurement of the Levels of Adherence to the Daily Food Guides

To improve the public’s knowledge of better diet options, in 1984, the Taiwan Department of Health (now Taiwan Ministry of Health and Welfare) collaborated with nutritionists and food personnel to establish the first edition of daily food guides [17]. The most updated daily food guides were released in 2018 and aimed to prevent nutrient deficiencies (70% Dietary Reference Intakes (DRIs)). In addition, daily food guides also considered evidence from epidemiologic studies to reduce the risk of cardiovascular and metabolic diseases. The daily food guides contain six food groups: (1) cereals and whole grains; (2) protein-rich foods (soybean, fish, egg, and meat); (3) dairy products; (4) vegetables; (5) fruits; (6) fats, oils, and nuts. The daily food guides recommend minimal servings for the above six food groups according to the individual daily energy needs. Dietary intake was assessed face-to-face by trained interviewers using a validated 79-item food-frequency questionnaire (FFQ) [11]. We have constructed a daily food guide index to measure the levels of adherence to the daily food guides. The components of the index, outlined in Table 1, were used to calculate a single score for each study participant, allowing for ranking of individuals according to their adherence to the daily food guides published in 2018 [18]. However, we did not assess the levels of adherence to fat and oil intakes because fat and oil intakes were not adequately assessed by FFQ.

For each participant, we estimated the appropriate quantity of each food group they should be consuming on the basis of their likely energy needs. This was calculated from healthy weight, resting metabolic rate, and level of physical activity [18,19]. The score that a person received in any of the food categories is determined by the appropriate number of servings for given daily energy needs (Table 1). A person who consumed the recommended number of servings from any food group would receive a score of 5 for the food group; conversely, a person consuming no serving within a food group would receive a score of 0. Between 0 and 5, the score is calculated proportionately; for example, a person who consumes three servings from protein-rich foods but he/she needed six, would be given a score of 2.5; for a person who consumes 2000 kcal/day to get the maximum score of 5 in the cereals and whole grains category, he/she needs to eat three servings of cereals and whole grains per day.

### 2.3. Statistical Analysis

Participants will be categorized by quartile of daily food guide index score. The associations of baseline personal characteristics with daily food guide index scores were evaluated using *t*-test for continuous variables and Chi-square or Fisher’s Exact tests for categorical variables. Confounding will be evaluated based on previous knowledge and taking into consideration the associations between daily food guide index scores with baseline characteristics and health outcomes.

Logistic regression models will be used to determine whether the daily food guide index score was related to the prevalence of metabolic syndrome (yes or no), as well as to each index of metabolic syndrome. Potential confounding factors will be selected based on prior knowledge and their relations with exposures and outcomes. All analyses will be performed by SAS 9.4 (SAS Institute, Cary, NC, USA).

## 3. Results

We divided the participants into two groups, participants with (n = 917) or without (n = 1617) metabolic syndrome. The demographic comparison between the two groups is shown in Table 2. Compared with participants without metabolic syndrome, participants with metabolic syndrome were more likely to be male (54% vs. 48%), to be older (age > 50: 79% vs. 51%), to have higher body mass index (BMI) (BMI >= 24: 80% vs. 32%), to have lower education level (College or above: 11% vs. 27%); to be married or live together (73% vs. 66%), and to have lower family income (Family income < New Taiwan dollar (NT) $40,000: 36% vs. 25%).

Table 3 displays the associations of adherence to overall daily food guide scores with the risk of having metabolic syndrome. After adjusting for age, sex, BMI, education level, marital status, and family income, we found negative associations between levels of adherence to daily food guides and metabolic syndrome. The odds ratios (ORs) for participants with the highest quartile of the adherence level was 0.65 (95% confidence interval (CI) = 0.48–0.88). Three indices of the metabolic syndrome were also negatively associated with the adherence levels. Participants with the highest quartile of the adherence levels had a lower risk of having abnormal waist circumference (≥ 90 cm for men and ≥ 80 cm for women). The OR was 0.62 (95% CI = 0.43–0.91); had a lower risk of having abnormal HDL-C (40 mg/dL for males 50 mg/dL for females). The OR was 0.75 (95% CI = 0.58–0.98) and had a lower risk of having abnormal triglyceride (≥ 150 mg/dL or medication use). The OR was 0.73 (95% CI = 0.55–0.97). We found no association between the adherence levels and the risk of having abnormal blood pressure and blood glucose.

Table 4 shows the associations between the levels of adherence to the individual food group index and the prevalence of metabolic syndrome. After adjusting for age, sex, BMI, education level, marital status, and family income, we found negative associations between the levels of adherence to food group 2 (scores ≥ 1) and the risk of having metabolic syndrome (OR: 0.60, 95% CI = 0.39–0.92), between the levels of adherence to food group 4 (scores ≥1) and the risk of having metabolic syndrome (OR: 0.57, 95% CI = 0.41–0.78), and between the levels of adherence to food group 5 (scores ≥ 1) and the risk of having metabolic syndrome (OR: 0.64, 95% CI = 0.45–0.92).

The associations between levels of adherence to overall daily food guides and demographic characteristics were shown in Table 5. Males were less likely to adhere to the daily food guides compared with females (OR: 0.34, 95% CI = 0.29–0.40); The younger the participants were, the less likely they adhered to the daily food guides. Participants who have lower education levels were less likely to adhere to the daily food guides. The OR for participants with elementary school or lower education was 0.21 (95% CI = 0.16–0.28), compared with those who had college or above education. Compared with those who were married, participants who were divorced, separated, widowed, or refused to answer were less likely to adhere to the daily food guides (OR: 0.34, 95% CI = 0.29–0.40). Participants who had lower family income were less likely to adhere to the daily food guides. Compared with participants with family income equal to or higher than NT $80,000, the OR for family income less than NT $10,000 was 0.63 (95% CI = 0.46-0.87). We found no association between the adherence levels and BMI.

## 4. Discussion

In this study, we found that participants reporting good adherence to daily food guides during the past month would have a lower risk of having metabolic syndrome. It seemed that the reduced risk was primarily from the reduced risk of having abnormal values of waist circumference, blood HDL-C, and blood triglyceride. No association was found between adherence to daily food guide and the risk of having abnormal blood pressure and blood sugar. We found a positive association between food group 1 (cereal and whole grains) and metabolic syndrome risk. Although it did not reach statistical significance, more studies are needed to examine whether high starch-containing food is recommended for preventing metabolic syndrome. It should be noted that we assumed that the participants’ food intake over the previous month reflects their long-term eating habits so that some correlations can be seen with metabolic syndrome. If the assumption was violated, some correlations between the adherence levels and metabolic syndrome might be weakened. In addition, good adherence to the guides of protein-rich food, fruit, or vegetable intakes, respectively, also were associated with reduced risk of having metabolic syndrome. We also found that males, younger age, lower education, divorced, separated or widowed, and with lower family income were associated with lower adherence to daily food guides.

Our findings were consistent with most previous studies. Several studies conducted in different countries found that fruit [20,21,22] or vegetable [23] intakes were associated with reduced risk of metabolic syndrome. Studies in Poland and Minnesota have also reported that the total intakes of fruit and vegetable were associated with decreased risk of metabolic syndrome [24,25]. A recent meta-analysis identified nine studies on fruit, nine on vegetables and seven on both fruit and vegetables and concluded that fruit, vegetables, or both were associated with reduced risk of metabolic syndrome [26]. The mechanism was not fully understood. It has been postulated that fruits and vegetables might reduce inflammatory markers [27], increase the dietary intake of foods high in dietary fiber and reduce those high in fat [26], or increase the intake of antioxidants such as vitamins A, C, and folate [28,29], which in turn reduce the risk of individual risk factors of metabolic syndrome. In addition, we found that good adherence to protein-rich food guides was associated with a reduced risk of metabolic syndrome. Although it involved only small numbers, a randomized controlled trial showed that heart healthy weight loss dietary patterns featuring foods rich in animal or plant protein improved aspects of metabolic syndrome [30].

The strengths of this study include a fair sample size to assess the possible association between adherence to daily food guides and the risk of having metabolic syndrome. Furthermore, the population-based design may allow us to generalize our findings to the public. The most important limitation of the study is its cross-sectional design, so the causality cannot be established. We only have a one-time assessment of the dietary assessment, which may introduce misclassification of exposures and outcomes. Another limitation is that we did not calculate the fats and oils intake because they were not adequately assessed by FFQ. How the fats and oils intake affected our findings was unclear. Some metabolic syndrome indices did not show statistically significant results. This may be due to looking at dietary patterns over a recent short period when the effects of dietary factors would have taken much longer to show effects. Their current dietary intake might not reflect past consumption patterns. We cannot rule out the possibility that the participants changed their dietary habits beyond our assessment period, and it might cause nondifferential misclassification of exposure. However, nondifferential misclassification of a categorical exposure usually biases toward the null. It tends to minimize the associations. In this situation, we still found protective effects between adherence to daily food guides and the risk of having metabolic syndrome, suggesting that the true effects might be underestimated.

In conclusion, the Taiwan daily food guides have the potential to help people reduce risk factors associated with metabolic syndrome, but longer and prospective studies are warranted to verify our findings.

## Figures and Tables

**Table 1:** Daily Food Guide for adults in Taiwan.

Index Item	Daily Energy Needs (kcal/day)						
	>=1200	>=1500	>1800	>=2000	>=2200	>=2500	>2700
Minimal servings from cereals and whole grains	1.5	2.5	3	3	3.5	4	4
Minimal servings from protein-rich foods	3	4	5	6	6	7	8
Minimal servings from dairy products	1.5	1.5	1.5	1.5	1.5	1.5	2
Minimal servings from vegetables	3	3	3	4	4	5	5
Minimal servings from fruits	2	2	2	3	3.5	4	4
Minimal servings from fats, oils, and nuts							
Fats and oils	3	3	4	5	5	6	7
Nuts	1	1	1	1	1	1	1

**Table 2:** Demographic description of included participants (N = 2534).

Variables	Without Mets	With Mets	
	*N* = 1617	*N* = 917	*p*-Value
**Sex**			
Males	751 (46%)	480 (52%)	0.0043
Females	866 (54%)	437 (48%)	
**Age**			
Age <= 30	312 (20%)	41 (5%)	<0.0001
40 => age > 30	231 (14%)	48 (5%)	
50 => age > 40	247 (15%)	104 (11%)	
65 => age > 50	469 (29%)	341 (37%)	
Age > 65	358 (22%)	383 (42%)	
**Body mass index**			
BMI < 24	1107 (68%)	186 (20%)	<0.0001
27 > BMI >= 24	333 (21%)	309 (34%)	
BMI >= 27	177 (11%)	422 (46%)	
**Education**			
Elementary school	852 (53%)	419 (46%)	<0.0001
Junior high and high school	322 (20%)	394 (43%)	
College or above	444 (27%)	104 (11%)	
**Marital status**			
Single	351 (22%)	70 (8%)	<0.0001
Married or lived together	1067 (66%)	669 (73%)	
Others	199 (12%)	178 (19%)	
**Family income**			
NT $10,000 > Income	100 (6%)	110 (12%)	<0.0001
NT $40,000 > Income >= NT $10,000	312 (19%)	222 (24%)	
NT $80,000 > Income >= NT $40,000	433 (27%)	195 (21%)	
Income >= NT $80,000	416 (26%)	168 (19%)	
Don’t know or refuse	356 (22%)	222 (24%)	

Mets: metabolic syndrome; NT: New Taiwan dollar.

**Table 3:** Associations of levels of adherence to overall daily food guide scores with the risk of having abnormal metabolic syndrome indices or metabolic syndrome.

	Levels of Adherence to Daily Food Guides			
	Q1 (*N* = 633)	Q2 (*N* = 634)	Q3 (*N* = 633)	Q4 (*N* = 634)
Metabolic Syndrome	Reference	0.69 (0.52–0.92)	0.52 (0.39–0.70)	0.65 (0.48–0.88)
Waist circumference	Reference	0.86 (0.59–1.24)	0.75 (0.52–1.08)	0.62 (0.43–0.91)
Blood pressure	Reference	1.15 (0.87–1.52)	0.87 (0.66–1.16)	1.11 (0.83–1.49)
HDL-C	Reference	0.79 (0.61–1.02)	0.74 (0.57–0.96)	0.75 (0.58–0.98)
Triglyceride	Reference	0.68 (0.52–0.88)	0.61 (0.46–0.80)	0.73 (0.55–0.97)
Blood glucose	Reference	0.86 (0.67–1.11)	0.80 (0.62–1.03)	0.91 (0.70–1.18)

Data were presented as odds ratio (95% confidence interval).

**Table 4:** Associations between the levels of adherence to individual food group index and the prevalence of metabolic syndrome.

Variables	*n* = 2534
**Food group 1 (cereals and whole grains)**	
Score < 0.5	Reference
0.5 <= Score <1	1.08 (0.74–1.59)
Score >= 1	1.34 (0.74–2.42)
**Food group 2 (protein-rich foods)**	
Score < 0.5	Reference
0.5 <= Score < 1	0.82 (0.66–1.02)
Score >= 1	0.60 (0.39–0.92)
**Food group 3 (dairy products)**	
Score < 0.5	Reference
0.5 <= Score < 1	0.87 (0.68–1.11)
Score >= 1	0.86 (0.46–1.62)
**Food group 4 (vegetables)**	
Score < 0.5	Reference
0.5 <= Score < 1	0.62 (0.45–0.84)
Score >= 1	0.57 (0.41–0.78)
**Food group 5 (fruits)**	
Score < 0.5	Reference
0.5 <= Score< 1	0.58 (0.37–0.91)
Score >= 1	0.64 (0.45–0.92)
**Food group 6 (nuts)**	
Score < 0.5	Reference
0.5 <= Score < 1	1.02 (0.66–1.56)
Score >= 1	0.87 (0.64–1.19)

Data were presented as odds ratio (95% confidence interval).

**Table 5:** The associations between levels of adherence to overall daily food guides and demographic characteristics.

Variables	*n* = 2534
**Sex**	
Females	Reference
Males	0.34 (0.29–0.40)
**Age**	
Age > 65	Reference
65 => age > 50	0.51 (0.41–0.62)
50 => age > 40	0.20 (0.15–0.27)
40 => age > 30	0.14 (0.10–0.19)
Age <= 30	0.12 (0.08–0.17)
**Body mass index (BMI)**	
BMI >= 27	Reference
27 > BMI >= 24	0.94 (0.77–1.16)
BMI < 24	0.90 (0.75–1.08)
**Education**	
College or above	Reference
Junior high and high school	0.56 (0.46–0.69)
Elementary school or lower	0.21 (0.16–0.28)
**Marital status**	
Married or lived together	Reference
Single	0.79 (0.60–1.05)
Divorced, separated, widowed, or refused to answer	0.62 (0.50–0.78)
**Family income**	
Income >= NT $80,000	Reference
NT $80,000 > Income >= NT $40,000	0.81 (0.65–0.99)
NT $40,000 > Income >= NT $10,000	0.60 (0.48–0.76)
NT $10,000 < Income	0.63 (0.46–0.87)
Don’t know or refuse	0.65 (0.52–0.82)

NT: New Taiwan dollar.
